# The Motivational Salience of Faces Is Related to Both Their Valence and Dominance

**DOI:** 10.1371/journal.pone.0161114

**Published:** 2016-08-11

**Authors:** Hongyi Wang, Amanda C. Hahn, Lisa M. DeBruine, Benedict C. Jones

**Affiliations:** Institute of Neuroscience and Psychology, University of Glasgow, Glasgow, United Kingdom; University Children's Hospital Tuebingen, GERMANY

## Abstract

Both behavioral and neural measures of the motivational salience of faces are positively correlated with their physical attractiveness. Whether physical characteristics other than attractiveness contribute to the motivational salience of faces is not known, however. Research with male macaques recently showed that more dominant macaques’ faces hold greater motivational salience. Here we investigated whether dominance also contributes to the motivational salience of faces in human participants. Principal component analysis of third-party ratings of faces for multiple traits revealed two orthogonal components. The first component (“valence”) was highly correlated with rated trustworthiness and attractiveness. The second component (“dominance”) was highly correlated with rated dominance and aggressiveness. Importantly, both components were positively and independently related to the motivational salience of faces, as assessed from responses on a standard key-press task. These results show that at least two dissociable components underpin the motivational salience of faces in humans and present new evidence for similarities in how humans and non-human primates respond to facial cues of dominance.

## Introduction

Multiple lines of evidence suggest that viewing attractive faces is rewarding [1–3]. For example, brain regions involved in the general processing of rewards, such as the nucleus accumbens and orbitofrontal cortex [4], respond more strongly when viewing physically attractive faces than they do when viewing physically unattractive faces [1,3]. Studies that have used key-press tasks to assess the motivational salience of faces (i.e., the extent to which participants will expend effort to alter the viewing time for a face) have also reported that participants will expend more effort to look longer at more attractive faces [5–8]. Some studies of heterosexual participants have reported that this effect of attractiveness on the motivational salience of faces is greater when viewing opposite-sex than own-sex faces [7,9], while others have reported this opposite-sex bias for male, but not female, participants [6], or have not observed an opposite-sex bias [8].

Whether physical characteristics other than attractiveness contribute to the motivational salience of faces is currently an unresolved issue. However, male macaques will exchange rewards to view dominant conspecifics’ faces, suggesting that more dominant-looking faces hold greater motivational salience for male macaques [10]. Given similarities in macaque and human face processing [11], this finding raises the possibility that dominance will also influence the motivational salience of faces in humans.

Recent work on the perceptual dimensions underlying social judgments of faces in humans suggests that social judgments of faces can be reduced to orthogonal valence and dominance components [12]. The valence component is highly correlated with traits such as perceived trustworthiness and attractiveness and appears to reflect perceptions of general prosociality [12]. The dominance component is highly correlated with traits such as perceived dominance and aggressiveness and appears to reflect perceptions of capacity to inflect physical harm [12]. Neurobiological evidence suggests that effects of attractiveness on neural markers of the motivational salience of faces may be better characterized as effects of the valence component than effects of attractiveness [1]. That male macaques find more dominant conspecifics’ faces more rewarding [10] suggests that the dominance component of social judgments of faces might also be associated with the motivational salience of faces in humans. This would be noteworthy because the motivational salience of faces is thought to drive the link between perceptual judgments and behavioral responses [5–7] and such results would suggest that the motivational salience of faces is not solely a consequence of their perceived valence.

The current study investigated whether the motivational salience of faces is positively and independently related to Oosterhof and Todorov’s [12] valence and dominance components. Motivational salience of faces was assessed using a standard key-press task used in many previous studies [5–8]. Responses to faces on this key-press task have been shown to predict neural markers of the reward value of faces [5]. Following Oosterhof and Todorov [12], principal component analysis was used to reduce ratings of faces on multiple traits to valence and dominance components.

## Method

### Face-rating task

Men (*N* = 260) and women (*N* = 260) participating in the face-rating part of the study (mean age = 22.97 years, SD = 5.52 years) were randomly allocated to rate either male or female faces for one of the 13 traits investigated by Oosterhof and Todorov [12] using 1 (low) to 7 (high) rating scales. All participants were between all between 16 and 40 years of age. These traits were aggressiveness, attractiveness, caringness, confidence, dominance, emotional stability, intelligence, meanness, responsibility, sociability, trustworthiness, unhappiness, weirdness. Ten men and 10 women rated each combination of trait and face sex and trial order within blocks was fully randomized.

**Fig 1:**
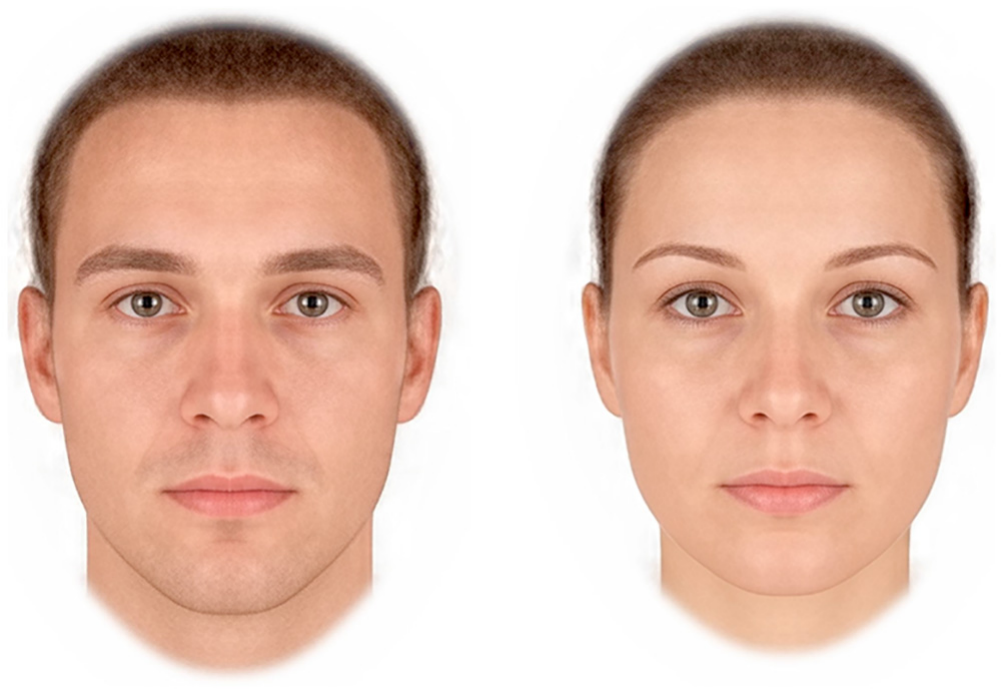
Prototypes with the average shape, color and texture information for the 50 male (left image) and 50 female (right image) faces used in the study. These are included as a representation of our stimuli only and were not used in our actual study.

Face stimuli were images of 50 white men (mean age = 24.2 years, SD = 3.99 years) and 50 white women (mean age = 24.3 years, SD = 4.01 years), posed front-on to the camera with direct gaze and neutral expressions to control for possible effects of gaze and emotion cues on reponses to faces. Images were aligned on pupil position and cropped so that clothing was not visible. The study was run online, with participants recruited from social bookmarking websites, such as stumbleupon.com. Fig 1 shows prototypes with the average shape, color and texture information for the 50 male and 50 female faces used in the study.

### Key-press task

A different set of 300 women (mean age = 21.77 years, SD = 4.15 years) and 300 men (mean age = 24.79 years, SD = 5.63 years) completed a standard key-press task, similar to those used to assess the motivational salience of faces in previous studies [5–8]. All participants were between 16 and 40 years of age. The same face images presented in the face rating part of the study were presented in the key-press task. Half the men and half the women were presented with images of the opposite-sex faces and the other half men and women were presented with images of the same-sex faces. Participants were randomly allocated to only one version of the task (i.e., saw either male faces or female faces). Trial order within each block was fully randomized. This part of the study was also run online. Online and laboratory studies of the motivational salience of faces have typically shown similar patterns of results [5–9].

In each version of the key-press task, participants controlled the viewing duration of each face image by repeatedly pressing designated keys on their keyboard after initiating each trial by pressing the space bar. Participants could increase the length of time a given face was displayed by alternately pressing the 7 and 8 keys and/or decrease the length of time a given face was displayed by alternately pressing the 1 and 2 keys. Each key press increased or decreased the viewing duration by 100ms. The default viewing duration for each image (i.e., the length of time a face remained onscreen if no keys were pressed) was 4s. Participants were told that the key-press task would last for a total of 3.5 minutes in order to discourage responses aimed at simply changing the length of engagement with the task. However, in reality, the total length of the key-press task was dependent on participants’ responses. All participants key-pressed at least once in the task. Participants completed a block of practice trials at the start of the test. Participants provided informed written consent before participating and University of Glasgow's School of Psychology Ethics Committee had approved all aspects of the study.

### Initial processing of data

Inter-rater agreement was high for all ratings (see Table 1), with the exception of unhappiness, for which inter-rater agreement was low for both male and female faces (both Cronbach’s alphas < .50). Consequently, unhappiness was discarded from the study. All other perceptual ratings were standardized within face sex (i.e., scores for male faces and scores for female faces were separately converted to z-scores) to control for possible effects of differences in how male and female faces were rated. Descriptive statistics for each trait are shown in Table 1, together with results of independent samples t-tests comparing ratings of male and female faces.

**Table 1:** Descriptive statistics for all traits considered in our analyses and results (t and p statistics) for independent samples t-tests for differences between ratings of male and female faces for each trait.

	Male faces			Female faces				
Trait	α	*M*	*SD*	α	*M*	*SD*	*t*	*p*
Aggressiveness	0.90	3.31	0.86	0.80	3.65	0.68	2.18	.032
Attractiveness	0.91	2.77	0.72	0.88	3.03	0.60	1.98	.051
Caringness	0.81	3.58	0.70	0.84	3.37	0.67	-1.52	.132
Confidence	0.86	3.87	0.69	0.85	3.71	0.72	-1.11	.272
Dominance	0.90	3.44	0.81	0.81	3.45	0.66	0.13	.897
Emotional stability	0.84	3.77	0.64	0.71	3.62	0.53	-1.25	.216
Intelligence	0.78	3.75	0.62	0.70	3.77	0.47	0.23	.821
Meanness	0.75	4.05	0.60	0.82	3.84	0.68	-1.56	.122
Responsibility	0.84	3.56	0.66	0.69	3.88	0.50	2.73	.008
Sociability	0.91	3.55	0.76	0.84	3.75	0.70	1.37	.173
Trustworthiness	0.84	3.34	0.61	0.77	3.90	0.56	4.73	<.001
Weirdness	0.90	4.49	0.83	0.74	4.25	0.58	-1.63	.106

*Note*. All variables were subsequently standardized within face sex.

Following previous studies of the motivational salience of faces [5–8], key-press scores for each face were calculated by subtracting the number of key presses made to decrease viewing time from those made to increase viewing time. These scores were calculated separately for each participant and served as the dependent variable in our analyses. Faces with greater key press scores are those with greater motivational salience [5]. Because inter-participant agreement in key-press scores for both male and female faces was high (both Cronbach’s alphas > .95), we calculated the average key-press score for each face. This was done separately for male participants (male faces: M = -6.04, SD = 2.96; female faces: M = -4.81, SD = 5.18) and female participants (male faces: M = -2.96, SD = 5.25; female faces: M = -3.00, SD = 4.03). As was the case for the perceptual ratings, these scores were standardized within face sex. Data are in the S1 File (Data used in analyses).

## Results

Following previous studies that used principal component analysis to reveal the components underlying ratings of social stimuli [12,13], we subjected all ratings to principal component analysis with no rotation. Two orthogonal components with eigenvalues greater than 1 were extracted. The first component explained approximately 50% of the variance in scores and was highly correlated with caringness, trustworthiness, and emotional stability. We labeled this the valence component. The second component explained approximately 24% of the variance in scores and was highly correlated with dominance and aggressiveness. We labeled this the dominance component. The component matrix is shown in Table 2. We used these components in our main analyses.

**Table 2:** Component matrix for principal component analysis of all traits.

Trait	Component 1 (valence)	Component (dominance)
Aggressiveness	-0.56	0.76
Attractiveness	0.78	0.36
Caringness	0.88	-0.26
Confidence	0.57	0.67
Dominance	-0.03	0.91
Emotional stability	0.86	0.13
Intelligence	0.65	0.27
Meanness	-0.59	0.74
Responsibility	0.71	0.22
Sociability	0.84	0.13
Trustworthiness	0.86	-0.27
Weirdness	-0.73	-0.20

Next, we analyzed key-press scores using ANCOVA with a custom model that included the within-items factor *participant sex* (male, female), the between-items factor *sex of face* (male, female), and scores on the *valence* and *dominance components* as covariates. The custom model included main effects of each factor and all possible two-way and three-way interactions, except ones including both the *valence* and *dominance components*.

This analysis revealed main effects of *valence* (F(1,94) = 105.00, p < .001, partial eta^2^ = .53) and *dominance* (F(1,94) = 17.10, p < .001, partial eta^2^ = .15). Faces that scored higher on the valence or dominance components generally had greater motivational salience (valence: r = .70, N = 100, p < .001; dominance: r = .28, N = 100, p = .004). Key-press score descriptive statistics for faces scoring ±1 SD from the mean on the valence and dominance components are given in Table 3. The correlation between valence and key-press scores was stronger than that between dominance and key-press scores (z = 3.82, p < .001, [14]). The interaction between *participant sex* and *valence* was not significant (F(1,94) = 3.25, p = .075, partial eta^2^ = .033). No other effects were significant or approached significance (all F< 1.53, all p> .22).

**Table 3:** Descriptive statistics of key-press scores for faces scoring ±1 SD from the mean on the valence and dominance components.

Component	Band	Mean	SD
valence	1 SD above the mean	0.39	4.30
valence	1 SD below the mean	-7.46	2.54
dominance	1 SD above the mean	-2.90	3.53
dominance	1 SD below the mean	-5.41	3.60

*Note*. that this table shows descriptive statistics for unstandardized key-press scores.

## Discussion

Principal component analysis of the initial face ratings produced two orthogonal components. Replicating previous research that has used this method to reveal the components that underpin social judgments of faces Oosterhof and Todorov [12], these components reflected the perceived valence and dominance of faces, respectively. Importantly, further analysis showed that both the valence and dominance components were positively and significantly correlated with the motivational salience of faces.

That faces scoring higher on the valence component had greater motivational salience is consistent with previous work reporting positive effects of attractiveness on the motivational salience of faces [6–8]. It is also consistent with neural evidence that overlapping brain networks drive the processing of facial attractiveness and facial trustworthiness [1].

Additionally, our analyses revealed systematic variation in the motivational salience of faces that was not due to valence, however. Faces that scored higher on the dominance component also had greater motivational salience. This effect of dominance complements results of studies of macaques, whereby male macaques were more willing to exchange juice rewards to view high-dominance, rather than low-dominance, conspecifics’ faces [10]. Positive correlations between facial dominance and cues of physical strength and aggression in humans have been widely reported [15]. Thus, greater motivational salience of more dominant faces may function, in part, to support the monitoring of individuals with high threat potential during social interactions. Note that, while male macaques were more willing to exchange juice rewards to view high-dominance faces [10], our participants showed smaller negative key-press scores for high-dominance faces, rather than larger positive key-press scores. Although it is tempting to interpret this pattern of results as indicating that high-dominance faces are less aversive, rather than more rewarding, to humans, this distinction between negative and positive key-press scores could simply reflect the length of the default viewing time (4s). Using a shorter default viewing time could reveal positive key-press scores for high-dominance faces.

Previous research has suggested that facial cues of dominance in conspecifics have similar effects on macaques’ and human’s responses to gaze-direction cues [16,17]. Our results linking dominance to the motivational salience of faces then present new evidence for similarities in human and macaque responses to facial dominance by extending results for motivational salience of facial cues of conspecifics’ dominance in macaques to human participants. Our face stimuli all had neutral expressions and direct gaze. Since emotional expressions and gaze direction can modulate responses to physical characteristics in faces [18–20], further work is needed to establish how these cues might modulate the motivational salience of valence and dominance.

## Supporting Information

S1 File Data used in analyses. (XLS)
